# Social patterns of miscarriage reporting and risk: insights from survey data in France

**DOI:** 10.1093/eurpub/ckaf099

**Published:** 2025-06-26

**Authors:** M C Compans, Heini Väisänen

**Affiliations:** Sexual and reproductive health and rights, Institut national d’études démographiques (INED), Aubervilliers, F-93300, France; Sexual and reproductive health and rights, Institut national d’études démographiques (INED), Aubervilliers, F-93300, France

## Abstract

Miscarriages, which are spontaneous pregnancy losses before 20–28 weeks of gestation, affect approximately 15% of recognized pregnancies. Existing population-based evidence of social inequalities in miscarriage risk is inconsistent, partly due to a lack of data. Surveys can be representative of a national population but are subject to underreporting of miscarriages. We examine whether miscarriages are underreported in a French nationally representative survey, FECOND (2010–11), and analyse socioeconomic risk factors for miscarriage. First, we apply a model that estimates miscarriage underreporting. Second, we use multilevel multinomial logistic regressions to examine socio-demographic and epidemiological factors associated with miscarriage. We estimate that 92% of miscarriages were reported, and underreporting was slightly more pronounced among lower-educated women. The estimated prevalence of miscarriages (14% of all pregnancies) is unaffected by underreporting rates and only with small educational differences. Thus, investigating social disparities in miscarriage risk can be done without correcting for reporting bias. Advanced reproductive ages are associated with higher miscarriage risk. Unobserved time-consistent individual characteristics explain the association between miscarriage risk and prior reproductive history. Conversely, education and self-assessed financial conditions are not associated with miscarriage risk. Younger cohorts exhibit a higher miscarriage risk, suggesting an age effect on recall, a cohort effect on pregnancy recognition, or reduced stigma resulting in more reliable reporting of miscarriage. In sum, the miscarriage reporting rate in FECOND survey is relatively high and with only slight social disparities. No large socioeconomic differences were found in miscarriage risk.

## Introduction

Miscarriages are spontaneous pregnancy losses before 20–28 weeks of gestation, with the definition varying depending on the context [1]. Around 13%–19% of medically recognized pregnancies [1] and 20%–30% of all pregnancies result in a miscarriage [2, 3].

Except for prospective clinical studies [3], all miscarriage data typically miss very early miscarriages [4]. Additionally, clinical studies tend to suffer from a lack of representativeness and small sample sizes. Health records typically only document miscarriages managed in hospitals and, less frequently, those handled by primary healthcare providers. Surveys can collect information on all pregnancies but are likely subject to misreporting (i.e. a respondent not reporting a pregnancy or mistakenly reporting its outcome). Without a gold standard, scholars have proposed model-based approaches to correct for misreporting of pregnancy outcomes, such as abortion [5].

While the extent of underreporting of abortions in high-income countries has been extensively studied with reporting rates ranging between 25% and 86% depending on survey and context [5–7], less is known about reporting of miscarriages. They may be less underreported than abortions because they can be more socially accepted [6, 8] but likely suffer from some underreporting in surveys [6, 8–11]. First, the underreporting of miscarriage may be due to miscarriages being omitted because of stigma, guilt, and emotional distress that can accompany such events [9]. This is supported by previous research showing face-to-face interviews prompt lower reporting rates than self-completion questionnaires [6], which may provide a greater sense of privacy and reduce the influence of stigma and emotional distress on reporting. This may be particularly true for low-income groups, resulting in social disparities in miscarriage reporting [6]. Second, a common source of underreporting in surveys collecting retrospective information is recall bias. Recall bias has been documented for various reproductive health outcomes [12], and miscarriages may be no exception to that, although one study in the USA suggests otherwise [13]. Third, early miscarriages can occur before the pregnancy is recognized by the pregnant person [4] (ciswomen, transmen, and non-binary individuals can experience miscarriages. In what follows, we refer to women based on the respondents’ self-identification, as it is often the only information available in quantitative data such as the one used in this paper). This is particularly likely when a pregnancy is unplanned and, as pregnancy planning often depends on socio-demographic characteristics, those with higher socioeconomic status might be more likely to detect their pregnancies earlier [14]. However, improvements in detecting early pregnancies through at-home pregnancy tests [15] may have increased awareness and reporting of miscarriages for younger cohorts. Pregnancies also tend to be recognized early when using infertility treatments, which is more common among more educated groups [16]. Lastly, miscarriage reporting can be influenced by the misclassification of induced abortions as spontaneous, particularly in contexts where abortions are not socially accepted [10]. Understanding the social patterns of miscarriage reporting is crucial for the estimation of social inequalities in miscarriage risks, as it may introduce bias in the results [17].

In addition, existing evidence on social inequalities in miscarriage risk is mixed. While some studies show lower education is associated with a higher miscarriage risk [18], others found an inverse relationship [19] or no association [20]. However, one could expect miscarriage risks to exhibit a social gradient, as observed in gestational diabetes, preterm birth, and neonatal death [21–23]. Socioeconomic status may be linked to a higher miscarriage risk via health-related factors. A range of specific health conditions, such as both low and high body mass index (BMI), have been shown to increase miscarriage risk [1, 24–26]. Because they tend to be more prevalent among socially disadvantaged groups [27], they potentially contribute to social disparities in the occurrence of miscarriages. Some studies also hint at the influence of the stability of the environment in which the pregnancy occurred. For instance, in the UK, experiencing a job loss during pregnancy increases the risk of pregnancy loss [28].

Against this backdrop, this paper first assesses whether patterns of reporting of pregnancies affect the analysis of miscarriage risk in the French FECOND survey conducted in 2010–11. To the best of our knowledge, this has rarely been done in previous miscarriage studies. Building on a modelling approach [5], we study underreporting of miscarriage with underreporting of abortion as a reference point. Second, we study socio-demographic and economic factors associated with miscarriage risk.

## Methods

### Data

The FECOND survey is a nationally representative study conducted in mainland France among 5272 women aged 15–49 in 2010–11. The interviews were conducted by phone with one individual per household using random dialling, with a response rate of 45% [29]. The survey investigates knowledge and practices related to sexual and reproductive health including a full pregnancy history. In this module, women were asked how many times they had been pregnant, regardless of the outcome of their pregnancies. The asked outcomes of each pregnancy included: ‘birth’, ‘abortion’, ‘miscarriage (blighted ovum)’, ‘ectopic pregnancy’, ‘medical termination’, or ‘stillbirth’ (authors’ translation). The questions for each pregnancy also included indicators of respondents’ socio-professional situation at the start of the pregnancy (see Table 1 for more information on variables used here).

**Table 1.** Description of individual and pregnancy characteristics in FECOND (2010–11)

	*n*	% (weighted with survey weights)
Age at the start of the pregnancy		
Below 20	428	8.5
20–24	1737	27.9
25–29	2705	36.3
30–34	1651	19.4
35–39	588	6.7
40 or over	87	1.1
Prior history of miscarriages		
None	5939	82.4
One miscarriage	990	13.6
Two or more miscarriages	267	4.0
Parity at the start of the pregnancy		
Childless	6017	81.8
One child	849	12.4
Two or more children	330	5.8
Financial situation at the start of the pregnancy		
No issues	3930	50.5
Somewhat tight	2357	34.7
Difficult	909	14.7
Relationship with the partner at the start of the pregnancy		
Stable	6245	85.8
Unstable	951	14.2
The pregnancy was causing issues with work		
No	6230	86.9
Yes	966	13.1
BMI at the time of survey		
Below 18.5	469	6.2
18.5–24.9	4746	62.6
25–29.9	1441	22.5
30 or over	540	8.7
Birth cohort		
1961–69	3300	46.8
1970–79	3232	42.4
1980–85	664	10.8
Education at the time of survey		
Below tertiary education	4078	72.9
Tertiary educated	3118	27.1
Born in mainland France		
Yes	6437	82.1
No	759	17.9
Importance of religion at the time of survey		
(Very) Important	1981	29.1
Not really important	2240	31.4
Not important	2975	39.5

### Estimation of miscarriage reporting

We first estimate the proportion of all pregnancies that ended in a miscarriage in FECOND overall and by education. This indicator is influenced by the extent to which pregnancies are underreported in the numerator as well as in the denominator. For this reason, we estimate the reporting rate of two potentially underreported outcomes: miscarriages and abortions.

We follow the modelling approach of Tennekoon [5], which addresses two key challenges in survey data: some respondents reporting zero events despite ever having experienced them (excess zeros), and others reporting a non-zero number but fewer events than actually experienced. To do so, the model employs a binomially-thinned zero-inflated Poisson framework that estimates the count and reporting rates of events. Thus, it is designed to analyse how many times an event has occurred. To account for the fact that some women report never experiencing an event despite having experienced it (zero-inflated Poisson mean) [2], the model equations read as follows:


$$\left\{ \begin{array}{c} P(Y=y\;|y>0)=\left(1-\varphi \right)\frac{{\left(\lambda t\right)}^{y}e^{-\lambda t}}{y!}(1)\\ P\left(Y=0\right)=\varphi +\left(1-\varphi \right)e^{-\lambda t}(2) \end{array}\right.$$

where Y is the count variable (pregnancy outcome), y is an integer above 0, and λ is the mean number of counts. t is the time of exposure, defined since the oldest event between the age of menarche or first sexual intercourse. λ=exp⁡(βX), with β a vector of estimated parameters and X a vector of covariates likely to influence the average count of events. $\varphi =\;\frac{\exp(\delta Z)}{1+\exp(\partial Z)}\;$is the probability that the outcome is equal to 0, with Z a vector of covariates likely to influence the zero-inflation process and δ the vector of corresponding coefficients.

Additionally, the model also accounts for the fact that people may report a non-negative count but lower than the actual number of events. It does so by estimating a probability π of events being correctly reported (reporting rates), which is incorporated with the Poisson distribution to adjust the estimated count by potential underreporting (binomially thinning). Let $Y^{*}$ be the true count:


(3) $$Y=\;\pi \;○\;Y^{*}=\;\pi \;○\;\frac{{\left(\lambda t\right)}^{y}e^{-\lambda t}}{y!}\;$$

where parameters are the same as in Equations (1) and (2). The model parameters are estimated with Maximum Likelihood Estimation (MLE) procedures.

Models were run on a sample of 4179 women. This is after the exclusion of 411 women who never had sexual intercourse, three women who only had same-sex intercourse and have never been pregnant, and six who had missing values on both ages at menarche and sexual intercourse. We further excluded 659 women with missing values on all the mentioned covariates below (up to 6% of missing values by covariate).

We adapted the Stata/Mata code provided by Tennekoon [5] to FECOND. For abortions, the model was first tested with the three equations including individual characteristics likely to influence the count of abortions, that is age at the time of the survey (15–25 years vs. 26–49 years), a dummy variable indicating whether religion is (very) important versus not important/no religion, a variable indicating whether women are tertiary educated or have a lower degree, a dummy variable indicating childlessness status, and whether women ever used contraception. Reporting (Equation (3)) also includes marital and employment statuses at the time of the survey, as those can influence the disclosure of abortions.

Contrary to induced abortions, miscarriages are unintentional events; hence, we used a slightly different set of control variables. The three model equations for miscarriages were first tested with a dummy variable for tertiary education, the age group, and parity at the time of the survey (childless, one child, or two children), whether women ever used infertility treatments, smoking as a dummy variable (having ever smoked), and BMI at the time of the survey as categorical (< 18.5 kg/m^2^, 18.5–24.9, 25–29.9, 30+). Reporting rates in Equation (3) are also estimated depending on marital status, self-assessed health status (very good or good vs. bad), and categories of net monthly income (< 1500€, 1500–2499€, 2500–3499€, 3500€ or over) at the time of the survey.

We started with models that included all potentially relevant variables. Then, we tested different model specifications by removing variables that prevented the models from converging. Finally, we retained the model with the best statistical fit based on the Akaike information criterion (AIC) and the Bayesian information criterion (BIC), which are presented in Supplementary Table S1 for miscarriages and Supplementary Table S3 for abortions. However, as highlighted in Tennekoon [5], estimates that rely on MLE procedures are sensitive to model specifications. Supplementary Table S4 displays average reporting rates obtained with alternative covariate combinations, although they do not exhibit the best model fit. These robustness checks yield average reporting rates ranging from 50% to 55% for abortions, suggesting robust findings. For miscarriages, the range is broader. For instance, when we exclude income, the estimated reporting rate (65%) is much lower than the 92% found in the three out of five models that include this variable (Supplementary Table S4).

### Estimation of miscarriage risk factors

To investigate factors associated with miscarriage risk, we select pregnancies reported by women respondents in FECOND who were at least 25 years old at the time of the survey. This age threshold ensures a reasonable exposure to pregnancy (there are only 123 pregnancies started before age 25). We employ a multilevel multinomial logistic regression with Bayesian inference. We model the three most common pregnancy outcomes: live birth, miscarriage, and abortion (7709 pregnancies after excluding 223 cases of other pregnancy outcomes). This way, we account for the fact that some women may decide to terminate pregnancies that would have resulted in a miscarriage [10, 30]. The model also accounts for an individual-level random effect to capture unobserved factors that may be correlated with the number of pregnancies a woman had and their outcomes. Let $Y_{ij}$ be the predicted outcome of pregnancy j for individual i and k=1,2,3 the type of pregnancy outcome. Using outcome 3 as the reference category (here, live birth):


$$P(Y_{ij}=k)=\frac{\exp(\eta_{\mathrm{ijk}})}{1+\exp\left(\eta_{ij1}\right)+\;\exp(\eta_{ij2})}$$

where $\eta_{ij3}=0$ (reference category) and for k=1,2:


$$\eta_{\mathrm{ijk}}=\beta_{0k}+\beta_{k}X_{ij}+u_{ik}$$

In this equation, $u_{ik}\;∼\;N({0,\sigma }_{u}^{2})$ captures the individual random effect and $\beta_{0k}$ is the intercept for outcome k. $X_{ij}$ is a set of individual and pregnancy predictors which includes the level of education, BMI at the time of the survey, birth cohort, country of birth, and importance of religion. For each pregnancy, models also control for several characteristics at the start of the pregnancy: age as categorical (< 20 and then 5-year age groups until 40+), prior history of miscarriages (none, one, or recurrent miscarriages), birth parity (childless, one child, two children, or more), the financial situation (no problems, a little tight, or difficult), and the stability of the relationship with the partner (stable or unstable). It also includes self-assessment of whether the pregnancy was causing difficulties with work. $\beta_{k}$ is a vector of corresponding estimated coefficients for outcome k.

The model is implemented using the *brms* package in R, which utilizes Hamiltonian Monte Carlo (HMC) via the programming language Stan for efficient posterior sampling [31]. After excluding 736 pregnancies with missing information on the explored variables (less than 4% for each covariate), models are run among 7196 pregnancies. They use default weakly informative priors. Four Markov chains are run with a total of 6000 iterations per chain. The distributions of the studied characteristics among this sample are displayed in Table 1.

## Results

### Miscarriage reporting

Applying Tennekoon’s [5] approach to miscarriage reporting, on average, 92% of miscarriages are estimated to be reported. While the level of education was not significantly linked with the number of miscarriages (Supplementary Table S1), it was negatively associated with the likelihood of reporting it. The model estimates that highly educated women report all their miscarriages, but the estimated reporting rate for lower-educated women is 85% (Table 2). In Table 2, low standard errors (0.02) are likely due to larger categories such as for the older age group, below tertiary educated women, and good health status (cf. Table 1). Women with good self-reported health status were less likely to report a miscarriage. At young ages, the reporting of miscarriages is particularly high in the young age group (25 or less at the time of pregnancy) and lower for women aged 26 or over.

**Table 2.** Estimated miscarriage reporting rates by socio-demographic characteristics in FECOND (2010–11)

	Reporting rate (%)	Standard error
Average	91.7	1.76
Age at the time of survey		
15–25 years old	100.0	0.21
26–49 years old	89.8	0.02
Parity at the time of survey		
Childless	99.0	0.19
One child	92.8	0.17
2+ children	85.8	0.05
Education at the time of survey		
< Tertiary education	84.8	0.02
Tertiary education	100.0	0.21
Marital status at the time of survey		
Married	87.6	0.09
Not married	94.6	0.12
Reported health at the time of survey		
Good	90.1	0.02
Not good	100.0	0.21
Monthly income at the time of survey		
< 1500€	97.5	0.18
1500–2499€	86.8	0.11
2500–3499€	87.1	0.13
3500+ €	98.6	0.20

The model applied to abortions in FECOND yields a 55% average reporting rate (Supplementary Table S2). This is within the range estimated by existing comparisons between FECOND and national statistics on abortions, which found a 66% reporting rate of induced abortions (95% confidence interval: [49%–89%]) [7]. We also find that there are no educational differences in reporting patterns of induced abortion by education (Supplementary Table S3).

When we correct for both the estimated reporting rate of abortions and miscarriages, we obtain a 13.9% miscarriage rate, which is similar to the reporting without correction (13.8%) (Table 3). With and without correcting for reporting biases, educational differences remain small.

**Table 3.** Pregnancy outcomes before and after correcting for estimated reporting rates of miscarriages and abortions in FECOND (2010–11)

	Reported in FECOND	Corrected by reporting rates for abortions and miscarriages	
	Pregnancy outcome (%)	Reporting rate (%)	Pregnancy outcome (%)
All women			
Miscarriages	13.9	92	13.8
Induced abortions	10.3	55	17.1
Live births	72.8	100	66.4
Other pregnancy losses	3.0	100	2.7
Tertiary educated			
Miscarriages	14.9	100	13.7
Induced abortions	10.3	55	17.3
Live births	71.6	100	66.0
Other pregnancy losses	3.2	100	3.0
< Tertiary educated			
Miscarriages	13.6	85	14.4
Induced abortions	10.3	55	16.9
Live births	73.2	100	66.1
Other pregnancy losses	2.9	100	2.6

Reporting rates have been estimated with binomially-thinned zero-inflated Poisson models [5].

Overall, this analysis suggests that FECOND is a reliable survey to study social inequalities in miscarriages. This allows us to investigate factors associated with miscarriages without additional correction for the reporting of pregnancy outcomes.

### Factors associated with miscarriage risk

**Figure 1.**
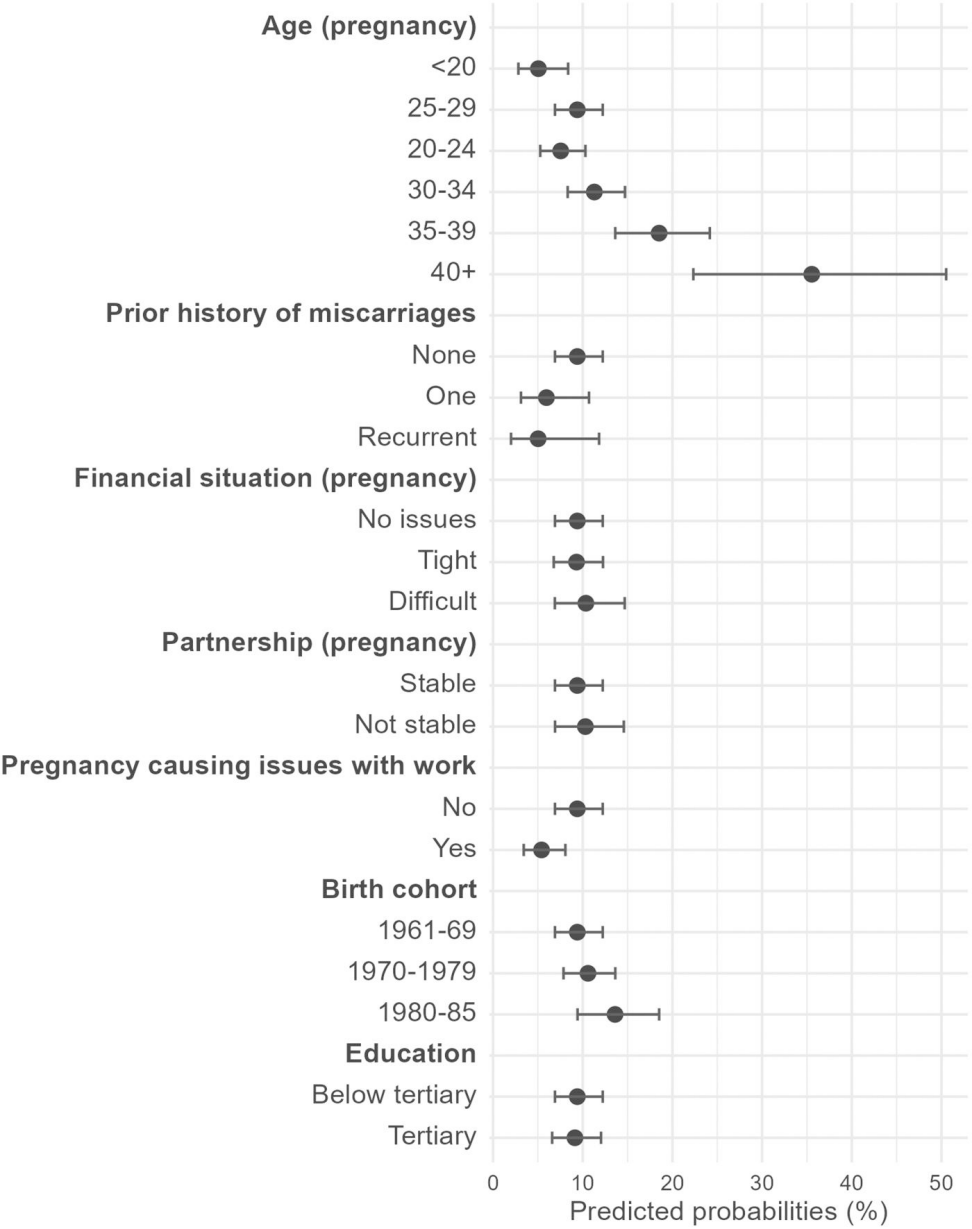
Predicted probabilities (and their confidence intervals at 95%) of pregnancies to result in a miscarriage by selected individual and pregnancy characteristics (multilevel multinomial model). Notes: full results shown in the Supplementary Table S5.

Figure 1 displays predicted probabilities of miscarriage risk associated with selected socio-demographic characteristics at the time of the survey (educational attainment) or at the time of each pregnancy (stability of the partnership, difficulties with work, financial situation). The full model, including estimates of abortion risk, can be found in Supplementary Table S5. Miscarriage risk does not significantly differ across educational groups. It significantly increases with age at conception. The predicted probability for a pregnancy to result in a miscarriage increases from 11.3% [8.3; 14.7] at age 30–34, to 18.5% [13.6; 24.2] at 35–39, and 35.5% [22.3; 50.5] at 40+. Compared to having no previous history of miscarriages, the predicted probability of losing the pregnancy is a bit lower for those who previously experienced miscarriage(s) and with large confidence intervals. Note that, in a model that does not account for a random effect, past history of pregnancy losses is positively associated with miscarriage risks (Supplementary Table S6), suggesting that time-invariant unobserved heterogeneity at the individual level influences the relationship. Pregnancies that ended in a miscarriage were less likely to involve work-related issues compared to those that resulted in live births, likely due to their shorter duration. Finally, younger cohorts of respondents (born in 1980–85) reported more miscarriages than older ones (13.6% [9.4; 18.5] vs. 9.4% [6.9; 12.2] for the 1961–69 cohort). Predicted probabilities do not vary much depending on self-assessed financial conditions and the stability of the partnership at the start of the pregnancy. The model also controls for parity at the start of pregnancy, BMI, religiosity, and country of birth, all of which have no significant association with miscarriage risk (Supplementary Table S5).

## Discussion

### Summary of the main results

Building on a model-based approach [5], we estimate that most miscarriages are reported (92%). We also find that 55% of induced abortions were reported, as in previous work [7]. It is in line with previous studies suggesting a greater disclosure of miscarriage compared to abortion [6, 8], although existing evidence for miscarriages is scarce. For instance, reporting rates have been found to be close to 60% for both miscarriages and abortions in the USA using the National Survey of Family Growth (NSFG) [11], or estimated between 50% and 80% in the World Fertility surveys in the Global South [10].

Correcting for abortion and miscarriage reporting rates, we estimate that 13.9% of recognized pregnancies result in a miscarriage. This is similar to estimates of miscarriage prevalence among clinically recognized pregnancies in other high-income countries [1, 15, 32, 33]. In addition, our model suggests that tertiary-educated women report all of their miscarriages, while the reporting rate is lower for lower-educated women (85%). Before and after correcting for this, educational differentials in miscarriage rates remain small, and thus, we did not correct for miscarriage or abortion reporting in our further analyses.

Our models reveal no significant differences in miscarriage risk by education, aligning with previous work in the UK [20], but contrasting with, for instance, analyses based on Danish registries, which found either a negative association [18] or a positive one with a larger sample and controls for childhood social conditions [19]. These inconsistencies may stem from either country differences or varying measurement designs, for instance, between using medical records limited to pregnancies that resulted in medical care rather than self-reported pregnancy outcomes. While disentangling contextual factors from methodological aspects remains challenging [7] and merits further investigation, social inequalities in miscarriage experience might also manifest in other ways, such as differences in miscarriage sequence (first vs. recurrent) or gestational timing (early vs. late pregnancy losses). Unfortunately, the limited sample size in our dataset does not allow us to explore this.

As shown previously [34], miscarriage risk increases with age at the start of the pregnancy. The positive association with prior history of miscarriages also found previously [34] does not hold when controlling for unobserved heterogeneity, as we did in our multilevel models. Instead, we find that pregnancies preceded by one previous miscarriage have slightly lower probabilities of resulting in another one. This may be due to random chromosomal abnormality resulting in a single pregnancy loss rather than systematic reproductive issues, leading these women to manage to achieve live birth in the next pregnancy [35].

Overall, both parts of the analysis show that younger respondents report more miscarriages. These patterns may reflect cohort differences in early recognition of pregnancy, along with improvements in available tests to detect it [15]. These differences could also stem from recall bias, presumably less severe for younger respondents, as shown for other reproductive health outcomes [12]. However, one study in the USA also suggests minimal recall bias, as the miscarriage prevalence measured for a given period remains consistent across subsequent cycles of the NSFG [13]. Alternatively, younger cohorts may perceive less stigma in disclosing miscarriages, which is worth exploring in future research.

### Strengths and limitations

One of the limitations in our study is that we assumed that pregnancy outcomes other than abortions and miscarriages are correctly reported in the survey, although ectopic pregnancies and stillbirths may also be misreported. However, as these events are relatively rare [36], their underreporting is unlikely to influence the denominator much in our calculation of miscarriage rates. Moreover, some of these pregnancy outcomes may be misreported as miscarriages, which can bias our estimation of miscarriage reporting rates upward. However, we do not know to what extent such mechanisms may apply. Future studies could investigate this with qualitative data.

Our analyses on how financial hardship at the time of the pregnancy is associated with miscarriage risk rely on retrospective information. Our findings differ from previous work showing a positive association between unemployment (which could be an indicator of financial hardship) and miscarriage risk [18, 28, 37]. However, unemployment may be a better indicator of socioeconomic conditions than the self-assessed financial situation we use.

Contrary to previous research, which measured BMI around the time of the pregnancy [1, 24–26], we find that BMI at the time of the survey is not significantly associated with miscarriage risk, but the results should be interpreted with caution. As BMI likely changes over the life course [38], measuring it at the time of survey may not accurately reflect the true association between BMI and miscarriage risk.

Despite these limitations, we show that survey data can capture pregnancy losses with reasonable accuracy within a nationally representative sample. Further research is still needed to confirm our findings, but at present, this study points to (1) a good reporting rate of miscarriages in FECOND, (2) only slight social disparities in miscarriage reporting and risks, and (3) time-invariant individual factors explain the role of previous miscarriage(s) on the risk of experiencing another one.

## Supplementary Material

ckaf099_Supplementary_Data

## Data Availability

This research uses data from the FECOND survey (Fertility, Contraception, and Sexual Dysfunction). Researchers interested in accessing these data can submit a request to PROGEDO-ADISP: https://www.progedo-adisp.fr/. Key points 92% of miscarriages are estimated to be reported in the French FECOND survey. With representative data, we estimate 14% of pregnancies to end in a miscarriage. We find no educational differences in miscarriage risk. Previous recurrent miscarriages influence miscarriage risk through unobserved heterogeneity.
