# Supplementary material for: Social patterns of miscarriage reporting and risk: insights from survey data in France
Source: Eur J Public Health. 2025 Jun 26;35(5):954–9. doi: 10.1093/eurpub/ckaf099 (PMC12529278; doi:10.1093/eurpub/ckaf099)
Supplement: Supplementary file 1 [file ckaf099_supplementary_data.zip › ejph-2025-02-om-0151-File003.odt]

Online supplementary material

Social patterns of miscarriage reporting and risk: Insights from survey data in France

Marie-Caroline Compans

Institut national d’études démographiques (INED)[^1]

Heini Väisänen

Institut national d’études démographiques (INED)[^2]

Table S1: Estimates of the count and reporting of miscarriages in FECOND (2010–11)

|  | Estimate | Standard error | p-value |
| --- | --- | --- | --- |
| Ever had a miscarriage |  |  |  |
| Intercept | 1.652 | 0.47 | <0.001 |
| 15–25 years old (ref = '26–49 years old') | 0.238 | 0.45 | 0.570 |
| Parity (ref='Childless') |  |  |  |
| One child | -1.285 | 0.48 | 0.006 |
| Two children or more | -1.757 | 0.37 | <0.001 |
| Ever smoked (ref= 'Never smoked') | -0.533 | 0.37 | 0.155 |
| < Tertiary education (ref = 'Tertiary education') | -0.429 | 0.37 | 0.187 |
| BMI (ref = '25.0–29.9') |  |  |  |
| Below 18.5 | 0.094 | 0.67 | 0.897 |
| 18.5–24.9 | 0.112 | 0.49 | 0.799 |
| 30 or over | -0.234 | 1.04 | 0.731 |
| Number of miscarriages |  |  |  |
| Intercept | -3.585 | 0.28 | <0.001 |
| 15–25 old (ref = '26–49 years old') | 0.728 | 0.29 | 0.077 |
| Parity (ref='Childless') |  |  |  |
| One child | 0.014 | 0.31 | 0.918 |
| Two children or more | -0.073 | 0.26 | 0.611 |
| < Tertiary education (ref = 'Tertiary education') | 0.104 | 0.21 | 0.740 |
| Ever been treated for infertility (ref= 'Never treated') | 0.777 | 0.14 | <0.001 |
| Ever smoked (ref= 'Never smoked') | -0.066 | 0.17 | 0.946 |
| BMI (ref = '25.0–29.9') |  |  |  |
| Below 18.5 | 0.029 | 0.32 | 0.973 |
| 18.5–24.9 | -0.071 | 0.22 | 0.754 |
| 30 or over | -0.443 | 0.42 | 0.210 |
| Reporting |  |  |  |
| Intercept | 32.605 | 4.12 | <0.001 |
| 15–25 old (ref = '26–49 years old') | 23.519 | 4.34 | 0.577 |
| Parity (ref='Childless') |  |  |  |
| One child | -1.234 | 3.32 | 0.899 |
| Two children or more | -1.800 | 3.39 | 0.714 |
| < Tertiary education | -13.359 | 2.96 | <0.001 |
| Married | -0.101 | 0.47 | 0.774 |
| Good reported health status | -17.174 | 1.08 | <0.001 |
| Monthly income (ref= '1,500–2,499 €') |  |  |  |
| Below 1,500 € | 2.000 | 1.94 | 0.390 |
| 2,500–3,499 € | -0.032 | 0.43 | 0.794 |
| 3,500 € or over | 2.002 | 2.20 | 0.684 |
| N | 4,179 |
| Log-likelihood | -2,472.810 |
| Akaike information criterion (AIC) | 5,001.619 |
| Bayesian information criterion (BIC) | 5,179.078 |

Note: Model developed by [5].  

Table S2: Estimated abortion reporting rates by women’s characteristics in FECOND (2010–11) 

|  | Estimated reporting rate (%) | Standard error |
| --- | --- | --- |
| Average | 54.6 | 0.12 |
| Age |  |  |
| 15–25 years old | 88.0 | 0.11 |
| 26–49 years old | 46.6 | 0.12 |
| Parental status |  |  |
| Childless | 69.2 | 0.11 |
| Parent | 46.7 | 0.11 |
| Education |  |  |
| Below tertiary | 50.0 | 0.11 |
| Tertiary | 60.0 | 0.11 |
| Importance of religion |  |  |
| (Very) important | 17.5 | 0.11 |
| Not important / no religion | - | - |
| Marital status |  |  |
| Married | 49.6 | 0.11 |
| Not married | 58.0 | 0.11 |
| Employment status |  |  |
| Employed | 57.0 | 0.12 |
| Not employed | 47.4 | 0.11 |

Note: Model developped by Tennekoon (2017).  

Table S3: Estimates of the count and reporting of induced abortions in FECOND (2010–11)  

|  | Estimate | Standard Error | p-value |
| --- | --- | --- | --- |
| Ever had an abortion |  |  |  |
| Intercept | 0.106 | 0.33 | 0.749 |
| Below tertiary education (ref = ‘Tertiary education’) | -0.580 | 0.41 | 0.159 |
| Religion is (very) important (ref = ‘Religion is not important / No religion’) | 0.608 | 0.41 | 0.134 |
| Never used contraception (ref = ‘Ever used contraception) | -2.599 | 12.21 | 0.832 |
| Number of induced abortions |  |  |  |
| Intercept | -3.559 | 0.25 | 0.000 |
| 15–25 years old (ref = '26–49 years old') | -0.551 | 0.39 | 0.157 |
| Childless (ref = ‘Mother’) | -0.256 | 0.24 | 0.294 |
| Below tertiary education (ref = ‘Tertiary educated’) | 0.193 | 0.23 | 0.396 |
| Religion is (very) important (ref = ‘Religion is not important / No religion’) | 2.187 | 0.56 | 0.000 |
| Never used contraception (ref = ‘Ever used contraception) | -2.489 | 1.33 | 0.062 |
| Reporting |  |  |  |
| Intercept | 1.780 | 0.97 | 0.065 |
| 15–25 years old (ref = '26–49 years old') | 3.813 | 1.26 | 0.003 |
| Childless (ref = ‘Mother’) | 0.243 | 0.68 | 0.721 |
| Below tertiary education (ref = ‘Tertiary educated’) | -0.663 | 0.51 | 0.194 |
| Religion is (very) important (ref = ‘Religion is not important / No religion’) | -2.686 | 0.64 | 0.000 |
| Married (ref = ‘Not married’) | -1.633 | 0.37 | 0.000 |
| Employed (ref = ‘Not employed’) | -0.555 | 0.24 | 0.018 |
| N | 4,179 |
| Log-likelihood | -2,023 |
| AIC | 4,081 |
| BIC | 4,188 |

Note: Model developped by Tennekoon (2017). 

Table S4: Average reporting rates obtained with various model specifications and model fit tests

|  | Average reporting rate | Akaike information criterion (AIC) | Bayesian information criterion (BIC) |
| --- | --- | --- | --- |
| Abortion |  |  |  |
| Final model (cf. Table 5) | 55 | 4,080.698 | 4,188.441 |
| Employment is not included in the reporting equation | 50 | 4,089.591 | 4,190.996 |
| Having ever been treated for infertility is included in the count and ever experiencing equations | 55 | 4,082.524 | 4,202.943 |
| Being partnered at the time of the survey is included in the reporting equation | 53 | 4,081.323 | 4,195.404 |
| Miscarriages |  |  |  |
| Final model (cf. Table 7) | 92 | 5,001.619 | 5,179.078 |
| Having ever been treated for infertility is included in the count and ever experiencing equations | 92 | 5,006.650 | 5,190.447 |
| Having ever used contraception is included in the count equation | 92 | 5,001.131 | 5,178.590 |
| Income is not included in the reporting equation and BMI is not included in the model | 79 | 5,000.629 | 5,127.386 |
| Income not included in the reporting equation | 65 | 5,010.064 | 5,168.510 |

Note: Model developped by Tennekoon (2017). 

Table S5: Estimates and confidence intervals at 95% (CI95) of factors associated with miscarriage and abortion risks vs. live birth (multilevel multinomial model) in FECOND (2010–11)

|  | Miscarriages |  | Abortions |
| --- | --- | --- | --- |
|  | Estimate | Standard Error | CI95 |  | Estimate | Standard Error | CI95 |
| Intercept | -2.26 | 0.16 | [-2.59;-1.96] |  | -4.48 | 0.24 | [-4.95;-4.04] |
| Age at the start of the pregnancy (ref='25–29') |  |  |  |  |  |  |  |
| Below 20 | -0.66 | 0.24 | [-1.14;-0.21] |  | 0.59 | 0.20 | [0.2;0.98] |
| 20–24 | -0.23 | 0.12 | [-0.46;-0.01] |  | 0.33 | 0.14 | [0.04;0.61] |
| 30–34 | 0.20 | 0.11 | [-0.01;0.42] |  | -0.35 | 0.17 | [-0.68;-0.03] |
| 35–39 | 0.79 | 0.16 | [0.48;1.11] |  | 0.19 | 0.21 | [-0.23;0.6] |
| 40 or over | 1.70 | 0.33 | [1.05;2.35] |  | 1.19 | 0.41 | [0.35;1.97] |
| Prior history of miscarriages (ref='None') |  |  |  |  |  |  |  |
| One miscarriage | -0.49 | 0.22 | [-0.94;-0.06] |  | -0.10 | 0.18 | [-0.45;0.24] |
| Recurrent miscarriages | -0.68 | 0.38 | [-1.41;0.07] |  | -1.02 | 0.45 | [-1.97;-0.19] |
| Parity at the start of the pregnancy (ref='Childless') |  |  |  |  |  |  |  |
| One child | 0.18 | 0.14 | [-0.1;0.45] |  | 0.97 | 0.17 | [0.64;1.32] |
| Two children or more | 0.45 | 0.22 | [0.01;0.9] |  | 1.81 | 0.25 | [1.31;2.31] |
| Financial situation at the start of the pregnancy (ref='No issue') |  |  |  |  |  |  |  |
| Tight | -0.01 | 0.09 | [-0.19;0.17] |  | 0.08 | 0.13 | [-0.18;0.34] |
| Difficult | 0.13 | 0.15 | [-0.17;0.43] |  | 1.19 | 0.15 | [0.9;1.48] |
| Unstable relationship with the partner at the start of the pregnancy (ref='Stable') | 0.21 | 0.14 | [-0.07;0.48] |  | 2.39 | 0.13 | [2.14;2.64] |
| The pregnancy was causing issues with work (ref='No issue') | -0.59 | 0.15 | [-0.88;-0.3] |  | 0.66 | 0.13 | [0.4;0.92] |
| BMI (ref=25.0–29.9') |  |  |  |  |  |  |  |
| Below 18.5 | -0.06 | 0.19 | [-0.44;0.31] |  | -0.10 | 0.23 | [-0.54;0.33] |
| 18.5–24.9 | -0.02 | 0.12 | [-0.25;0.21] |  | -0.15 | 0.15 | [-0.44;0.13] |
| 30 or over | 0.04 | 0.18 | [-0.31;0.4] |  | -0.61 | 0.24 | [-1.1;-0.15] |
| Birth cohort (ref='1961–69') |  |  |  |  |  |  |  |
| 1970–79 | 0.14 | 0.1 | [-0.07;0.34] |  | 0.23 | 0.13 | [-0.02;0.48] |
| 1980–85 | 0.43 | 0.17 | [0.1;0.76] |  | 0.57 | 0.19 | [0.2;0.94] |
| Tertiary education (ref='Below tertiary education') | -0.02 | 0.1 | [-0.22;0.17] |  | 0.49 | 0.12 | [0.26;0.72] |
| Not born on the mainland territory (ref='Yes') | -0.01 | 0.16 | [-0.32;0.29] |  | 0.59 | 0.17 | [0.24;0.92] |
| Importance of religion (ref='Not really') |  |  |  |  |  |  |  |
| (Very) Important | -0.14 | 0.12 | [-0.38;0.09] |  | -0.29 | 0.16 | [-0.61;0.03] |
| Not important | -0.06 | 0.11 | [-0.27;0.15] |  | 0.50 | 0.14 | [0.24;0.78] |
| N | 7,196 |
| Watanabe-Akaike information criterion (WAIC) | 8,513 |

Table S6: Estimates and confidence intervals at 95% (CI95) of factors associated with miscarriage and abortion risks vs. live birth (multinomial model) in FECOND (2010–11)

|  | Miscarriages |  | Abortions |
| --- | --- | --- | --- |
|  | Estimate | Standard Error | CI95 |  | Estimate | Standard Error | CI95 |
| Intercept | -1.88 | 0.10 | [-2.09;-1.68] |  | -3.96 | 0.17 | [-4.29;-3.63] |
| Age at the start of the pregnancy (ref='25–29') |  |  |  |  |  |  |  |
| Below 20 | -0.47 | 0.20 | [-0.87;-0.08] |  | 0.47 | 0.18 | [0.13;0.82] |
| 20–24 | -0.18 | 0.10 | [-0.38;0.02] |  | 0.26 | 0.13 | [0.01;0.52] |
| 30–34 | 0.13 | 0.09 | [-0.05;0.31] |  | -0.31 | 0.15 | [-0.6;-0.01] |
| 35–39 | 0.59 | 0.12 | [0.34;0.83] |  | 0.25 | 0.19 | [-0.13;0.63] |
| 40 or over | 1.32 | 0.26 | [0.81;1.83] |  | 1.09 | 0.37 | [0.38;1.81] |
| Prior history of miscarriages (ref='None') |  |  |  |  |  |  |  |
| One miscarriage | 0.32 | 0.09 | [0.13;0.5] |  | -0.13 | 0.16 | [-0.44;0.18] |
| Recurrent miscarriages | 0.85 | 0.15 | [0.57;1.13] |  | -0.92 | 0.40 | [-1.71;-0.13] |
| Parity at the start of the pregnancy (ref='Childless') |  |  |  |  |  |  |  |
| One child | -0.05 | 0.11 | [-0.27;0.17] |  | 0.76 | 0.15 | [0.47;1.05] |
| Two children or more | 0.03 | 0.17 | [-0.3;0.35] |  | 1.36 | 0.20 | [0.97;1.75] |
| Financial situation at the start of the pregnancy (ref='No issue') |  |  |  |  |  |  |  |
| Tight | -0.01 | 0.08 | [-0.16;0.15] |  | 0.05 | 0.12 | [-0.18;0.29] |
| Difficult | 0.14 | 0.13 | [-0.11;0.4] |  | 1.04 | 0.13 | [0.78;1.29] |
| Unstable relationship with the partner at the start of the pregnancy (ref='Stable') | -0.43 | 0.12 | [-0.68;-0.19] |  | 2.19 | 0.10 | [1.99;2.4] |
| The pregnancy was causing issues with work (ref='No issue') | -0.43 | 0.12 | [-0.16;0.15] |  | 0.58 | 0.12 | [0.35;0.81] |
| BMI (ref=25.0–29.9') |  |  |  |  |  |  |  |
| Below 18.5 | -0.03 | 0.14 | [-0.32;0.25] |  | -0.10 | 0.20 | [-0.48;0.29] |
| 18.5–24.9 | -0.01 | 0.09 | [-0.19;0.17] |  | 0.52 | 0.15 | [0.23;0.81] |
| 30 or over | -0.02 | 0.13 | [-0.28;0.25] |  | 0.21 | 0.11 | [0;0.42] |
| Birth cohort (ref='1961–69') |  |  |  |  |  |  |  |
| 1970–79 | 0.10 | 0.08 | [-0.05;0.24] |  | 0.21 | 0.11 | [0;0.42] |
| 1980–85 | 0.33 | 0.13 | [0.08;0.59] |  | 0.54 | 0.16 | [0.22;0.85] |
| Tertiary education (ref='Below tertiary education') | 0.00 | 0.07 | [0.07;0.07] |  | 0.44 | 0.10 | [0.1;0.1] |
| Not born on the mainland territory (ref='Yes') | 0.03 | 0.12 | [-0.21;0.26] |  | 0.52 | 0.15 | [0.23;0.81] |
| Importance of religion (ref='Not really') |  |  |  |  |  |  |  |
| (Very) Important | -0.12 | 0.09 | [0.08;0.59] |  | -0.24 | 0.14 | [-0.51;0.04] |
| Not important | -0.05 | 0.08 | [-0.3;0.06] |  | 0.46 | 0.12 | [0.23;0.7] |
| N | 7,196 |
| Akaike information criterion (AIC) | 8,677 |

[^1]: F-93300 Aubervilliers, France, [marie-caroline.compans@ined.fr](mailto:marie-caroline.compans@ined.fr) (corresponding author)
[^2]: F-93300 Aubervilliers, France, [heini.vaisanen@ined.fr](mailto:heini.vaisanen@ined.fr)
